# Frontier model of the environmental inefficiency effects on livestock bioeconomy

**DOI:** 10.12688/f1000research.128071.1

**Published:** 2022-11-25

**Authors:** C. A. Zuniga Gonzalez, J. L. Jaramillo-Villanueva

**Affiliations:** 1 Agroecology Department, National Autonomous University of Nicaragua, Leon, Leon, 21000, Nicaragua; 2 Colegio de Posgraduados, Puebla, 72760, Mexico

**Keywords:** Stochastic Frontier, Solar Activity, Technical Efficiency, Livestock Bioeconomy

## Abstract

**Background:** This work was focused on measuring environmental inefficiency in Mexican dairy farms, considering climate change variables related to the emission of greenhouse gases (GHG) and planetary geomagnetic activity.

**Methods:** The applied methodology measures the eco-efficiency of Mexican dairy farms using the empirical application of a stochastic frontier model of the bioeconomy. The productive sector of the bioeconomy studied was the eco-intensification of the livestock production system (dairies). The environmental inefficiency effect was assumed to be a distribution-independent truncation of a normal distribution with constant variance, while the mean was a linear environmental function of the observable variable.

**Results:** The results showed that the coefficients of the frontier model were highly significant, highlighting the investment in livestock (50%). The inefficiency model had an impact on climate variation with greenhouse gas emissions CH4 (1.96%). The results of the environmental technical efficiency in geometric average were 81.28%. The producers that reached the border with a technical efficiency equal to 1 are the references for the rest, marking the relative technical efficiency.

**Conclusions:** It was concluded that the coefficients in the model were very significant, showing the level of investment in livestock (50%). The low-performance model estimates the impact of climate change on GHG emissions CH4 (1.96%) explaining the trend of increasing GHG emissions, keeping in view that the management of food and cattle during the study period were affected by summer feeding, which allowed considering the activity of GHG emissions. According to the results, the geometric mean environmental performance of engineering is 81.28%.

## 1. Introduction

Livestock has historically been considered one of the most important activities in Latin America given its great influence on the economy. It is for this reason that the livestock subsector has been a pivotal axis for the various studies that evaluate performance, and it is here that the analysis of technical inefficiency becomes important for this type of study. In such a way that the need to be more efficient is not a recent discussion, but has been a concern of our predecessors in terms of production, it is thus that the governments intend to introduce a series of strategies in the different rural development plans, which consider the transformation of livestock systems from extensive to intensive. Pérez *et al*. (2004) assessed that in Latin America, livestock activity ranks seventh in world production and tenth in milk production. In 2001, it contributed about 4.7% of total world meat production and 0.17% of milk. Latin America has great resource potential, however, it has not been possible to meet the demand for milk and meat, so this type of study was necessary to determine the efficiency of dual-purpose production systems. For their part, Morillo and Urdaneta (1998) focused that the proportion of income derived from the sale of milk as against the sale of animals for meat varies greatly from 12% to 80%, depending mainly on the producer's objectives, of the growth phase in which the males are sold and of the racial types, in any case, influenced by the agro-ecological characteristics of the farms and the technology used.

According to the diagnosis of the Sectoral Program for Agricultural, Fisheries and Food Development 2013—2018 of Mexico in 2050, the world population will be 9,300 million people and the Food and Agricultural Organization (FAO) estimates that the world demand for food will increase by 60% ( FAO, 2012). For that year, the population in Mexico will grow by 34 million, reaching 151 million people. The sustained growth of some developing countries such as Brazil, China, and India poses challenges and opportunities worldwide for the development of the agri-food sector. The International Monetary Fund (IMF) estimates the growth of the world economy at 3.8% annual average for the next six years, with important differences between the groups of countries; 5.2% for emerging markets and 2.2% for advanced economies, which will lead to increases in food consumption globally . This trend represents an important opportunity for Mexico to perform an important lead in meeting world food needs. However, arable land is limited both in the world and in Mexico. It is necessary to face climate change which translates into extreme weather events that affect food production. In this context, the great global challenge is to increase food production through higher productivity ( López-González *et al.,* 2016a, 2016b).

In Mexico, climate change has manifested itself in unprecedented and unexpected extreme events. In 2009, the worst drought in 60 years occurred; 2010 was the wettest year on record; and in 2011, there were intense and atypical frosts, and less rainfall. In September 2013, heavy rains occurred that caused some damage to agriculture and, unfortunately, loss of human life. In several parts of the country, for a few days it only rained being comparable to half of all that rained in 2012. The consequences of these natural phenomena are reflected in the loss of part of the production, outbreak of diseases, and lower levels of income and wealth for the population. The Mexican Climate Modeling Network developed an ensemble of projections that represents the country's climatology considering the various climatic scenarios. There is concern in Mexico that in the coming decades, the temperature will increase more than the historical average, that is, 6% higher than the global increase. ( Maza *et al.,* 2017; Milán and Zúniga-Gonzalez 2021; Núñez and Montalvo 2015).

Historical facts have confirmed this temperature rise. Therefore, an increase in the danger of climatic events associated with increased warming or a decrease in crop yield can be expected, even if they have not been recorded historically. Most graphical representations of rainfall, tropical cyclones, northerly winds, and cyclones do not include the degree of uncertainty. The way of producing food is changing; Technological innovation, infrastructure, organization of productive activities, sustainable practices, and risk management in primary activities are the main public policy instruments to achieve greater resilience in the agri-food sector. It is in this context that the study of the effects of inefficiency in livestock production systems is worthwhile as part of the livestock bioeconomy of the eco-intensification production path ( Zuniga-Gonzalez *et al.,* 2014; Dios-Palomares *et al.,* 2015a: Dios-Palomares *et al.,* 2015b; Dios-Palomares, 2002; Morillo, 1998).

This work was organized with a section that refers to the literature review of the technical efficiency model, a third section was dedicated to the evaluation of the empirical application of the model, and later the results, discussion, and conclusions were presented.

## 2. Literature review

### Frontier model of the effects of environmental inefficiency on livestock bioeconomy

The study considered the environmental stochastic frontier adjusted to the livestock bioeconomy. In the equation below

$$Y_{\mathrm{it}}=\exp\left(X_{\mathrm{it}}\beta +\nu_{\mathrm{it}}-\nu \mu_{\mathrm{it}}\right)$$ (1)

$Y_{\mathrm{it}}$ denotes the production of milk and its derivatives in the dairy farms in the study area in the *t-th* observation regarding the time ( *t =*1,2, … …… …, T) and on the *i-th* sample *(i = 1,2,3 … ……………… ….N);*

$X_{\mathrm{it}}$ is a vector (1 x k) of known values of the productive climatic parameters (input of milk production and measurement parameters of greenhouse gases (GHG)). These explanatory variables were associated with the *i-th* sampling point *y* on the *t-th time* observed *;*

β is a vector (k × 1) of unknown parameters to be estimated; $V_{\mathrm{it}}$ was assumed to be an identical and independent distribution ( *dii*) of random errors $\;N\;\left(0,V_{\nu }^{2}\right)$ , distributed independently $U_{\mathrm{it}}$ ;

$U_{\mathrm{it}}$ were non-negative arbitrary variables, related to the technical inefficiency of production (milk as a production system in the livestock bioeconomy), which are assumed to be independently distributed. These $U_{\mathrm{it}}$ were obtained by truncation (at zero) of the normal distribution, $z_{\mathrm{it}}\;\delta$ , y variance, $\sigma^{2}$ ;

$z_{\mathrm{it}}$ was a vector (1 x m) of explanatory variables related to the technical inefficiency of the sampling points completed time; y δ , is a vector (m x 1) of unknown coefficients to be estimated.

Then, equation (1) specifies the environmental function for a productive sector of the livestock bioeconomy, about the data of the livestock bioeconomy system in livestock farm systems. However, for the effects of environmental technical inefficiency, the $U_{\mathrm{it}}$ was expected to be a relation of the set of independent variables, and the $z_{\mathrm{it}}$ was a vector of unknown coefficients, δ . The independent variables in the environmental technical inefficiency model may include approximately stochastic frontier components, although this is not our case, indicating that the effects of environmental inefficiency were stochastic. If the value of the first z-variables was 1 and the coefficients of the other z *-*variables was zero, then this case represents the model specified by Stevenson (1980) and Battese and Coelli (1988, 1992) ^*^. When δ-vector was equal to zero, inefficiency effects were unrelated to the z-variables, resulting in the mean normal distribution, originally specified in Aigner, Lovell, and Schmit (1977), obtained. If the interaction between the variables of the specific sampling points and the input variables of the livestock bioeconomy system was of the z-variables, then non-neutral probability limits, proposed by Huang and Liu (1994), would be obtained.

The effects of technical inefficiency, $U_{\mathrm{it}}\;$ , in the stochastic frontier model (e. 1) could be specified in an equation 2:

$$U_{\mathrm{it}}=z_{\mathrm{it}}\delta +W_{\mathrm{it}}$$ (2)

where $W_{\mathrm{it}}$ is a random variable defined by the truncation of the normal distribution with zero mean and variance, $\sigma^{2}$ , such that the truncation point is $-z_{\mathrm{it}}\;\delta ,i.e.,W_{\mathrm{it}}\geq z_{\mathrm{it}}\;\delta .$ These conventions were reliable with $U_{\mathrm{it}}$ presence of a truncation of the distribution $N\;\left(z_{\mathrm{it}}\delta ,\sigma^{2}\right)$ not negative. The inefficiency frontier production function represented in eq. 1 and 2 differs from Reifschneider and Stevenson (1991) in that the random variables were not uniformly dispersed or were not obligatory to be negative. In addition, average, $z_{\mathrm{it}}\delta$ , of the normal distribution is truncated at zero to obtain the distribution $U_{\mathrm{it}}$ and it does not require to be positive for each observation, as in Reifschneider and Stevenson (1991).

The supposition that $U_{\mathrm{it}}$ and $V_{\mathrm{it}}$ are distributed self-sufficiently despite the *t=* 1.2.3 … …, T, y *i*=1,2,3, … ………………… …..,N, was simplified, but with limiting condition. Substitute models were essential for an explanation of the possible correlated structures of the effects of technical inefficiency and arbitrary errors at the frontier.

The maximum probability technique (maximum likelihood) was proposed for simultaneous estimations of the stochastic frontier parameters and the model of technical inefficiency effects. The probability function and its partial derivations concerning the model parameters are presented by Battese and Coelli (1993). The probability function is expressed in terms of the variance parameters $\sigma_{s}^{2}\equiv \sigma_{\nu }^{2}+\sigma^{2}\;y\;\gamma \equiv \frac{\sigma^{2}}{\sigma_{s}^{2}}$ .

The technical efficiency of milk production for the *t=* 1.2.3 … …, T, and *i*=1,2,3, … ………………… …..,N is defined in equation 3:

$${\mathrm{ET}}_{\mathrm{it}}=\exp\left(-U_{\mathrm{it}}\right)=\exp\left(-z_{\mathrm{it}}\delta -W_{\mathrm{it}}\right)$$ (3)

The forecast of the environmental technical efficiency is based on its conditional expectancy, given the assumptions of the model. This result is also given in Battese and Coelli, (1993).

## 3. Methods

The work was carried out in the state of Tlaxcala, which is located in the Mexican Altiplano and at the geographic coordinate’s 98°43” west longitude and 19°44' north latitude, and 97°38' east longitude, 19° north latitude and 06 south latitude. The prevailing climate in the state is sub-humid temperate with summer rains. The average altitude of the study region is 2,200 meters above sea level.

For the collection of data, the following procedure was followed: a) identification of the areas of the state with the highest volume of milk production, b) identification of the production units present in the study areas, c) design and application of a questionnaire to collect information, and d) analysis of the data obtained. The study was carried out in 102 cattle farms for milk production in six municipalities of the state, in 2020. The production units were randomly selected, and divided into four regions of importance in dairy production in the state of Tlaxcala. The questionnaires contained technical information, owner information and economic data. In addition, 102 dairy cattle farms were monitored.

### Variables

The methodology used was known as the stochastic production frontier, which is based on the Cobb-Douglas function ( Battese & Coelli, 1992 and 1995). This is an empirical application of the Battese and Coelli (1995) model.

The FRONTIER (RRID:SCR_022958) Version 4.1 computer program ( Battese & Coelli, 1988, 1992 and 1995) was used to obtain a maximum likelihood estimate (MLE) of the selected data in the study period; this is raised in the literature review section. The model used based on eq. 1, is the following eq. 4:

Stochastic frontier model for a livestock bioeconomy system.

$$\ln\left({\mathrm{TVA}}_{\mathrm{ij}}\right)=\beta_{0}+\beta_{1}\;\ln\;\left({\mathrm{CIG}}_{\mathrm{ij}}\right)+\beta_{2}\;\ln\;\left({\mathrm{CT}}_{\mathrm{ij}}\right)+\beta_{3}\ln\;{\left(\mathrm{MO}\right)}_{\mathrm{ij}}+\beta_{4}\ln{\left(\mathrm{ND}\right)}_{\mathrm{iJ}}+\beta_{5}\ln{\left(\mathrm{EP}\right)}_{\mathrm{iJ}}+\left(\nu_{i}-\mu_{i}\right)$$ (4)

where

( ${\mathrm{TVA}}_{i}$ ) represents the total annual sale of products obtained on the farm, such as the amount of milk produced per cow per year and by secondary products. The unit of measure is in dollars.

( ${\mathrm{CIG}}_{\mathrm{ij}}$ ) represents the annual value of the investment quantified in dollars.

( ${\mathrm{CT}}_{\mathrm{ij}}$ ) represents the total annual cost for fuel, feeding, reproduction, illness and treatment, milking, mortality, and preventive medicine, measured in annual dollars.

( ${\mathrm{MO}}_{\mathrm{ij}}$ ) represents the annual cost of family and hired labor, measured in dollars.

(SG) surface destined for livestock (Ha)

( ${\mathrm{ND}}_{\mathrm{ij}}$ ) represents the number of dependents measured in people.

( ${\mathrm{EP}}_{\mathrm{ij}}$ ) represents the age of the producer measured in years of age.

( $\nu_{i}$ ─ $\mu_{i}$ ), the compound error component $\nu_{i}$ represents arbitrary variables that were assumed to be normally distributed in N (0, $\sigma_{v}^{2}$ ) and independent of $\mu_{i}$ , represents non-negative arbitrary variables that were assumed to measure technical inefficiency in production, γ is assumed to be independently distributed as zero truncations of the normal distribution N( $\omega_{\mathrm{it}}$ , γ) equation 2. These measurements are interpreted as indicators of the relative importance of each variable in the composition of the compound error in such a way that if gamma takes a value close to 1, it follows that there are no effects on the error due to factors beyond the control of the body of the area studied ( Dios-Palomares *et al.,* 2002; Dios-Palomares *et al.,* 2015).

(i) represents the period in which the samples were taken: 02/05/2020 to 30/10/2022.

(j) represents the sample of 102 farms in our case,

Model of technical production inefficiency for a livestock bioeconomy system: environmental condition and variability in climate change.

$$U_{\mathrm{ij}}=\delta_{0}+\delta_{3}\left(N{\mathrm{EP}}_{\mathrm{ij}}\right)+\delta_{2}\left({\mathrm{CAE}}_{\mathrm{ij}}\right)+\delta_{3}\left(\mathrm{CH}4_{\mathrm{ij}}\right)+\omega_{i}$$ (5)

The effects of environmental inefficiency in the study region are assumed to be defined by eq 5.

where $U_{\mathrm{ij}}$ was error term that measures the environmental technical inefficiency effect in the Mexican region of study considering the variability of climate change, explained in the previous section.

( ${\mathrm{NEP}}_{\mathrm{ij}}$ ) represents educational level of the producer.

( ${\mathrm{CAE}}_{\mathrm{ij}}$ ) represents the amount of water used per animal unit, measured in liters.

( $\mathrm{CH}4_{\mathrm{ij}}$ ) represents greenhouse gas emission of methane from enteric fermentation measured in Gg CH4/year.

( $\omega_{\mathrm{ij}}$ ) is the random variable explained in the previous section.

Hypothesis to be tested: If the inefficiency model is stochastic, then the technical efficiency of the dairy farm system can be explained by the Stochastic Frontier model for a livestock bioeconomy system influenced by climate change variability (greenhouse gas emissions).

Table 1 shows the statistical description of the data used in this study. The full protocol can be found on protocols.io.

**Table 1.** Descriptive statistics.

Variables	N	Minimum	Maximum	Mean	Std. Deviation
Total income $ (TVA_ij)	102.00	0.00	156,103,200.00	3,851,211.69	18,148,341.49
Investment cost in livestock ($) (CIG_ij)	102.00	16,000.00	38,826,000.00	1,028,312.75	4,401,851.61
Total annual cost for feeding, reproduction, diseases and treatments, preventive medicine, sanitation, milking, fuel (CT_ij)	102.00	7,300.00	44,020,690.00	1,029,632.72	4,942,898.56
Total M/O (MO_ij)	102.00	43,800.00	2,701,000.00	235,168.33	377,025.73
Area destined for livestock (Ha) (SG)	102.00	0.00	260.00	14.84	38.64
Number of dependents (people) (ND_ij)	102.00	1.00	13.00	5.31	2.26
Producer age (years) (EP_ij)	102.00	18.00	77.00	46.29	12.54
Educational level (years) (NEP_ij)	102.00	0.00	19.00	9.13	3.69
Amount of water used per animal unit, measured in liters (CAE_ij)	102.00	2,111.19	67,803.82	22,157.53	10,597.10
Greenhouse gas emissions Methane from enteric fermentation measured in Gg CH4/year. (CH4_ij) *	102.00	112.00	224,672.00	5,915.14	25,701.35

Note:

* Emission factor established by the 2006 IPCC for Latin America ( Herranz-Ramirez, 2018).

Source: Author’ self-calculation

## 4. Results

### 4.1 Empirical Analysis

The estimates with standard error parameters of the maximum likelihood (maximum-likelihood) are calculated with two significant digits, as shown below, according to eq. 4 and 5, respectively, in the conditions of milk-producing farms:

Stochastic frontier livestock bioeconomy model:

$$\ln\left(\mathrm{TVA}\right)=-1.94+0.59\ln\left(\mathrm{CIG}\right)+0.26\ln\left(\mathrm{CT}\right)+0.30\;\ln\left(\mathrm{MO}\right)+0.002\;\left(\mathrm{SG}\right)-0.24\ln\left(\mathrm{ND}\right)+0.25\ln\left(\mathrm{EP}\right)$$

$$\;\left(1.09\right)\;\left(0.09\right)\;\left(0.07\right)\;\left(0.09\right)\;\left(0.05\right)\;\left(0.14\right)\;\left(0.17\right)$$

Inefficiency model of livestock bioeconomy:

$$U=1.2-0.36\left(\mathrm{NEP}\right)-0.27\left(\mathrm{CAE}\right)+1.96\left(\mathrm{CH}4\right)$$

$$\;\left(0.89\right)\;\left(0.09\right)\;\left(0.02\right)\;\left(0.89\right)$$

Variance of Parameters:

$$\sigma_{s}^{2}=0.23,\gamma =0.0003$$

$$\left(0.29\right)\;\left(0.0003\right)$$

$$\mathrm{Log}\;\left(\text{likelihood}\right)=-71.74$$

**Figure 1.**
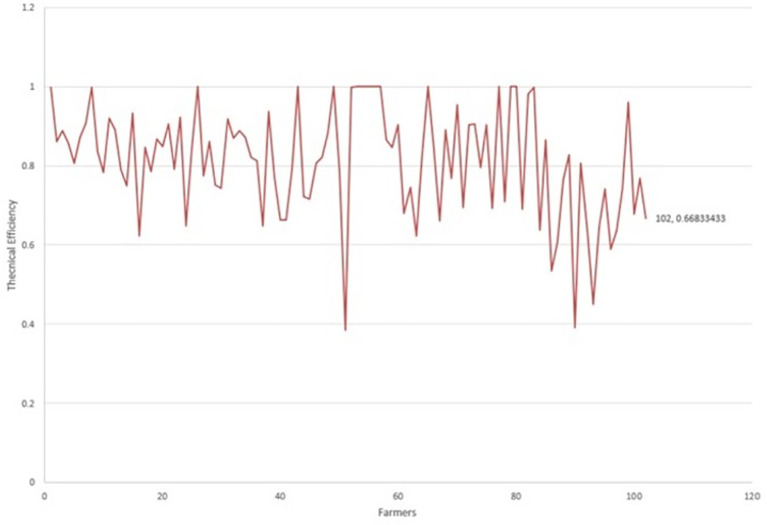
Technical efficiency per dairy farm.

The coefficient signs of the environmental stochastic frontiers of the livestock bioeconomy model were as expected. The negative elasticity of the model in dairy farms is interpreted as a non-scale economy that depends fundamentally on variations in the cost of investment in cattle, labor, the number of dependents, the total cost, the surface used in cattle farming and the age of the producer to ensure good quality milk. These coefficients were highly significant, highlighting the investment in livestock (50%). The inefficiency model was of particular interest in this study. The impact of climatic variation with GHG emissions CH4 (1.96%) explains a tendency to increase GHG emissions, considering that the feeding and management of livestock in the study period were affected by summer feeding, which allows us to consider the GHG emission activity. The result of the environmental technical efficiency in geometric average was 81.28%. Figure 1 presents the behavior of the environmental technical efficiency indices in terms of the quality of dairy production. The producers that reached the border with a technical efficiency equal to 1 are the references for the rest, marking relative technical efficiency.

The estimate in the variance parameters, σ _s_^2^, was close to one (0.23), implying that the quality of milk production was highly significant. It was generalized that the effects of the inefficiency in the null hypothesis likelihood-ratio test were absent or had the simplest distribution, (see Table 1). The first null hypothesis pointed out that the effects of inefficiency were absent from modeling, and was therefore, strongly rejected. The second null hypothesis, specifying non-stochastic inefficiency effects, was also strongly rejected. The third null hypothesis, considered in Table 2, specifies that the effects of inefficiency were not a linear function for the educational level, nor of the amount of water used, nor GHG emissions. This null hypothesis has also been rejected at the 5% significance level. This indicates that the stated effects of these three explanatory variables on inefficiency in dairy farms were significant. The effects of inefficiency for the stochastic frontier were clearly stochastic and were not related to the observations of the educational level, amount of water used, and GHG emissions. Thus, the stochastic frontier environmental inefficiency function was an improvement over the environmental stochastic frontier suggested by Dios Palomares *et al.* (2015b), Zúniga-González *et al.* (2022).

**Table 2.** Hypothesis test for the parameters of the frontier model of environmental inefficiency for the farms studied.

Null Hypothesis	Log (likelihood)	$\chi_{0.95}^{2}-\text{valor}$ ^†^	Statistics Test	Decision
${Η}_{0}=\gamma =\delta_{0}=\ldots \delta_{4}=0$	78.77	9.48	14.05 *	Rechaza H _0_
${Η}_{0}=\gamma =0$	-1.93	7.85	36.29 ^*^	Rechaza H _0_
${Η}_{0}=\delta_{1}=\delta_{2}=\delta_{3}=0$	71.44	7.85	14.05 *	Rechaza H _0_

^†^ The likelihood-ratio statistical test, λ= -2{log [likelihood (H0)] - [log [likelihood (H1)]} has about an x-distribution with estimators equal to the number of estimators assumed to be zero in the null hypothesis, H _0_; subsequently H _1_ is true. If the estimator, *γ*, is zero, then the variances in the inefficiency effects are zero and so the model reduces to the traditional mean response function. In this case, the estimators, δ0 and δ1, are not defined. Henceforth, the critical value for the statistical test for this second hypothesis was obtained from the χ _1_^2^ distribution.

* One asterisk in the estimate of the statistical test indicated that it exceeds the 95th percentile for the corresponding Chi-square distribution (χ ^2^) and consequently the null hypothesis was rejected.

## 5. Discussion

A stochastic frontier model of environmental inefficiency effects was proposed for dairy farms in Mexico Zúniga-González *et al.* (2014), under environmental conditions, following Dios Palomares *et al*. (2015), Rangel Cura *et al.* (2015). An application of the model was presented using data from 102 dairy farms. The results indicated that the model for the of environmental efficiency effects, involved a constant term, investment costs in livestock, total annual costs for feeding, labor, area for livestock, number of dependents, and the producer age, which was a significant component in the environmental stochastic frontier function. Model specification allowed the estimation of both changes and the variation of the GHG emission as environmental inefficiency effects, given that the effects of inefficiency were stochastic and had an unknown distribution. In addition, theoretical and applied work was required in the paths of bioeconomy to obtain better and more generalized stochastic frontier models and environmental inefficiency effects associated with the analysis of Battese & Coelli (1995), Trigo *et al.* (2015), Dios Palomares *et al.* (2015), Dios Palomares *et al*. (2015b).

In the geometric average, the environmental technical efficiency for variable climate conditions was 89%, which represents a regular quality of water and is strongly explained by the decreasing trend or inelasticity of solar activity. We add that during the months of the study, the variability of the geomagnetic activity was low, making it necessary to include data where the variations represent geomagnetic storms that would imply strong variations. Regarding the political agenda, the study shows the need to promote bioeconomy in the productive paths of eco intensification, biotechnology, and biorefineries, mainly to treat the waste generated by agricultural activities, mines, and livestock, Colon-García *et al.* (2021), Catari-Yujra *et al.* (2022), García-Bucio *et al.* (2022), Fernández-Santos *et al.* (2013). Referring to the management of GHG emissions both in enteric fermentation and waste management is very important in dairy production. These regulations must be aimed at setting emission standards (discharge limits) with alternatives for residual use with bioeconomic goods and the establishment of quality objectives ( González-Araya & Vásquez, 2010; Zúniga-González *et al*., 2022; Georgescu-Roegen, 1976; Kuramoto, 2021).

## Conclusion

This research used Stochastic Frontier Analysis (SFA) to study the determinants of the frontier efficiency model of dairy farms in the state of Tlaxcala in 2020 using a frontier model of environmental inefficiencies on the livestock bioeconomy.

The results were estimated by maximum likelihood. First, the frontier model was completed and the second was a bioeconomically inefficient livestock model. These coefficients were very significant, showing the level of investment in livestock (50%). The low-performance model estimates the impact of climate change on GHG emissions CH4 (1.96%) explaining the trend of increasing GHG emissions, keeping in view that the management of food and cattle during the study period were affected by summer feeding, which allowed considering the activity of GHG emissions. According to the results, the geometric mean environmental performance of engineering is 81.28%.

## Data Availability

Figshare: DataSFA.csv. figshare. Dataset. https://doi.org/10.6084/m9.figshare.21434343.v2 ( Zúniga-González & Jaramillo-Villanueva, 2022). This project contains the following underlying data:
‐ DataSFA.csv Data are available under the terms of the Creative Commons Attribution 4.0 International license (CC-BY 4.0).

## Footnotes

^*^
The intercept $\delta_{0}$ was not added as an estimator, in the middle $z_{\mathrm{it}}.\delta$ could result for the δ estimators related to the z variables with the trend and shape of the distribution of the effects of technical inefficiency, U_it, being unnecessarily constrained.
