# Supplementary material for: Frontier model of the environmental inefficiency effects on livestock bioeconomy
Source: F1000Res. 2024 Jun 18;11:1382. Originally published 2022 Nov 25. [Version 3] doi: 10.12688/f1000research.128071.3 (PMC11344204; doi:10.12688/f1000research.128071.3)
Supplement: Peer review history

# Reviewer report: Reviewer response for version 3

**DOI:** 10.5256/f1000research.167735.r292318

**Published:** 2024-09-24

**Authors:** Jakub Staniszewski

**Affiliations:** 1 Uniwersytet Ekonomiczny w Poznianiu, Poznań, Poland

Although the authors have addressed most of my comments, in many cases the changes made to the text are not sufficient. I will insist that the paper does not estimate environmental inefficiency, but the environmental determinants of economic efficiency. Also, the use of family size (ND) and farmer age (EP) as factors of production seems to me unjustified. Rather, these are environmental variables determining the level of inefficiency. I believe that without a recalculation the model is not suitable for indexing. I therefore continue to recommend major revision.

Is the work clearly and accurately presented and does it cite the current literature?

No

If applicable, is the statistical analysis and its interpretation appropriate?

No

Are all the source data underlying the results available to ensure full reproducibility?

Yes

Is the study design appropriate and is the work technically sound?

No

Are the conclusions drawn adequately supported by the results?

No

Are sufficient details of methods and analysis provided to allow replication by others?

Yes

Reviewer Expertise:

environmentally adjusted efficiency analysis

I confirm that I have read this submission and believe that I have an appropriate level of expertise to state that I do not consider it to be of an acceptable scientific standard, for reasons outlined above.

# Reviewer report: Reviewer response for version 3

**DOI:** 10.5256/f1000research.167735.r310761

**Published:** 2024-08-24

**Authors:** Jorge Leon Quiroga-Canaviri

**Affiliations:** 1 Universidad Mayor de San Andres, La Paz, La Paz Department, Bolivia

**Argumentation:**

 Dear authors. The article is interesting and the econometrics used seem appropriate to me, therefore, I recommend its indexing for replication in other countries, understanding that the problem described is current and the analysis formulated is pertinent.

**Methodologically:**

 I consider that the model used and the bibliographic support presented are sufficient for its exploratory development. The quotes are explanatory and help the reader understand how and why the data processing was carried out.

 The novelty of the manuscript is the climatic treatment, considering the environmental component, which according to my experience is innovative, although it could deserve some sophistications that are not necessarily necessary to be carried out in this proposal, which is still exploratory.

**Relevant positive aspects:**

 Truncation of the normal distribution refers to the modification of the normal distribution in such a way that values in a specific range are eliminated or restricted. This occurs when the data set is limited to a certain interval, which may occur for practical reasons, such as the impossibility of observing or recording certain values, which we believe to be the reason for the authors.

 For example, if we consider a variable that follows a normal distribution, truncation can occur if only values greater than a certain threshold are observed, thus eliminating all values below that threshold. This action changes the statistical properties of the distribution, affecting its mean, variance, and other characteristics.

 In econometrics, truncation can be relevant in regression models and data analysis, as it can influence results and statistical inference.

 Recommendation: As a recommendation, it is necessary to take into account that truncation can introduce biases if the information is not properly handled, so specific methods should be considered to deal with truncated data, such as truncated regression models. These models are designed to correct for bias that can result from truncation and provide more precise estimates of model parameters and not necessarily for missing information.

**Recommendation:**

 Nicholas Georgescu-Roegen is cited in the modeling, using a Cobb Douglas function, to run the model. Georgescu-Roegen clearly and openly opposed the linear analysis introduced by Cobb Douglas functions; see [ref 1] and [ref 2]. I suggest to explain and include this argument in the paper.

Is the work clearly and accurately presented and does it cite the current literature?

Yes

If applicable, is the statistical analysis and its interpretation appropriate?

Yes

Are all the source data underlying the results available to ensure full reproducibility?

Yes

Is the study design appropriate and is the work technically sound?

Yes

Are the conclusions drawn adequately supported by the results?

Yes

Are sufficient details of methods and analysis provided to allow replication by others?

Yes

Reviewer Expertise:

Reviewer's experience: Expert in Bioeconomy and econometric analysis.

I confirm that I have read this submission and believe that I have an appropriate level of expertise to confirm that it is of an acceptable scientific standard.

## References

[1] : From Bioeconomics to Degrowth Georgescu-Roegen's 'New Economics' in Eight Essays. *Routledge Taylor and Francis Group* .2014;

[2] : Brief exposition of Georgescu Roegen's contributions to ecological economics and a critical commentary. *New Era* .2008;

# Reviewer report: Reviewer response for version 3

**DOI:** 10.5256/f1000research.167735.r310759

**Published:** 2024-08-23

**Authors:** Sergio Gabriel Ceballos-Pérez

**Affiliations:** 1 National Council for Humanities, Science and Technology, Ciudad de México, Mexico

The article shows the environmental impact of intensive livestock farming in some parts of Mexico, with a sample to generate an efficiency model, particularly of greenhouse gas emissions and production inputs. The stochastic frontier model is based on the principles of technical efficiency (TE) and resource optimization [ref 1]. The doubts that arise throughout the article are due, from a particular point of view to factors that need to be explained, from the theoretical and practical part of the frontier models, how they have been used, what they are used for, as well as the adaptation that was made to introduce an environmental variable. Please review the work of [ref 1] and [ref 2], they explain the model and theory in a simple way.

 It is necessary to work on the wording and make it more understandable for the reader, from the summary, the methodology, the results, and the conclusions.

 In the background section, there is a statement that causes noise, if the phrase “planetary geomagnetic activity” were removed because it has nothing to do with the article, the work would improve greatly.

 In the methodological part, it is mentioned how the information was obtained, however, it would be good to clarify some doubts such as: How many bovine production units are there in Tlaxcala? How many of them are dairy farms? since it is mentioned that 102 were chosen randomly, but the universe is not mentioned. It would also be good to specify what information was collected in the questionnaire or make a summary table of the data obtained from the questionnaires.

 Explain the results, discussion, and conclusions better, so they are clearer.

 Include more updated references at least halfway through the article.

Is the work clearly and accurately presented and does it cite the current literature?

Partly

If applicable, is the statistical analysis and its interpretation appropriate?

I cannot comment. A qualified statistician is required.

Are all the source data underlying the results available to ensure full reproducibility?

Partly

Is the study design appropriate and is the work technically sound?

Yes

Are the conclusions drawn adequately supported by the results?

Yes

Are sufficient details of methods and analysis provided to allow replication by others?

Partly

Reviewer Expertise:

Bioeconomy, circular economy, environmental Kuznets curve, environmental accounts.

I confirm that I have read this submission and believe that I have an appropriate level of expertise to confirm that it is of an acceptable scientific standard.

## References

[1] : El efecto de las subvenciones en la ganadería española: un análisis mediante un modelo de frontera estocástica. *Pecvnia : Revista de la Facultad de Ciencias Económicas y Empresariales, Universidad de León* .2012; 10.18002/pec.v0i14.594 10.18002/pec.v0i14.594

[2] : Mediciones de eficiencias en sistemas silvo pastoriles. *Horizontes Empresariales* .2022;21(2) : 10.22320/hem.v21i2.5795 20-41 10.22320/hem.v21i2.5795

# Response

**Published:** 2024-08-25

**Authors:** C. A. Zuniga-Gonzalez

**Affiliations:** Agroecology. ECAV. CIByCC., National Autonomous University of Nicaragua, Leon. Bioeconomy and Climate Change Research Center., Leon, Leon, Nicaragua

Dear Reviewer,

 Thank you for your thoughtful feedback and detailed review of our manuscript. We appreciate the time you have taken to provide your insights.

 We would like to address the points you raised and clarify that the issues mentioned have already been discussed in the manuscript:

1. **Explanation of Frontier Models and Environmental Variables**: The theoretical and practical aspects of frontier models, including their application and adaptation to environmental variables, are thoroughly discussed in the relevant sections of the manuscript. We have detailed the stochastic frontier model, its purpose, and its integration with environmental considerations, as outlined in the methodology and discussion sections.
2. **Readability**: We have worked to ensure that the summary, methodology, results, and conclusions sections are presented clearly and are understandable. We believe these sections address the need for clarity and accessibility for the reader.
3. **Background Section**: The background section has been carefully reviewed and revised to focus on the relevant topics. The phrase “planetary geomagnetic activity” has been discussed and its relevance has been clarified in the context of the article's focus.
4. **Methodological Details**: We have provided detailed information on the number of bovine production units in Tlaxcala, the proportion of dairy farms, and the sampling process in the methodology section. Additionally, the data collected from the questionnaires is summarized in a table for better understanding (see Data availability).
5. **Results, Discussion, and Conclusions**: The results, discussion, and conclusions sections have been crafted to clearly explain the findings and their implications. We believe these sections adequately address the interpretation of the results and their relevance.
6. **References**: We have included relevant references throughout the manuscript to ensure that the discussion is supported by up-to-date research.

We hope this clarification demonstrates that the points you raised have been addressed within the manuscript. Thank you again for your valuable feedback.

 Best regards,

 Carlos

# Reviewer report: Reviewer response for version 3

**DOI:** 10.5256/f1000research.167735.r310758

**Published:** 2024-08-22

**Authors:** Morantes Martiña

**Affiliations:** 1 Instituto-Departamento de Producción Animal, Facultad de Agronomía, Universidad Central de Venezuela, Caracas, Capital District, Venezuela

This work represents a significant contribution to the study of milk production systems in America, where the availability of data sets for efficiency analysis is limited. Based on the approach of this work, the application of the stochastic frontier model is in accordance with the stated objectives. To improve the support of this article, the following is suggested:

 It is necessary to clarify the methodology for selecting the sample of farms: for example, the level of production of milk to be selected as milk producers and not dual-purpose cattle.

 It is necessary to indicate the software used for descriptive statistics. And to explain, based on bibliographic support, the methodology for the selection of environmental variables and their incorporation into the model.

 You must improve the discussion, it is necessary to explain the characteristics of dairy systems because it would allow researchers from other latitudes to have a comprehensive vision of how these systems are in terms of management, ecological conditions, level of production, etc.

 It is important to describe how water consumption and greenhouse gases were calculated.

 In the discussion, it is recommended to describe the management of grazing, forage resources, and breeds, among other characteristics, which is essential to explaining the results in the inefficiency model.

Is the work clearly and accurately presented and does it cite the current literature?

Yes

If applicable, is the statistical analysis and its interpretation appropriate?

Yes

Are all the source data underlying the results available to ensure full reproducibility?

Partly

Is the study design appropriate and is the work technically sound?

Yes

Are the conclusions drawn adequately supported by the results?

Yes

Are sufficient details of methods and analysis provided to allow replication by others?

Partly

Reviewer Expertise:

Animal Production Sytems

I confirm that I have read this submission and believe that I have an appropriate level of expertise to confirm that it is of an acceptable scientific standard.

# Response

**Published:** 2024-08-22

**Authors:** C. A. Zuniga-Gonzalez

**Affiliations:** Agroecology. ECAV. CIByCC., National Autonomous University of Nicaragua, Leon. Bioeconomy and Climate Change Research Center., Leon, Leon, Nicaragua

Dear Reviewer,

 Thank you for your insightful feedback on our study. We appreciate your recognition of the contribution our work makes to the analysis of milk production systems in America.

 We will address your comments as follows in our revised manuscript:

1. **Sample Selection Methodology:** We will clarify in the methodology section that our sample consists of dairy farms rather than dual-purpose cattle. Additionally, the introduction specifies that the study area is dedicated to dairy production, which aligns with our focus on milk-producing farms.
2. **Software and Methodology for Descriptive Statistics:** We will specify that the software used for descriptive statistics is The FRONTIER (RRID
  ) Version 4.1 computer program (Battese & Coelli, 1988, 1992, and 1995) as outlined in the methods section of our manuscript.
3. **Discussion of Dairy Systems:** Details about the dairy systems, including management practices, ecological conditions, and production levels, are comprehensively explained in the protocol repositories (protocols.io) and the DATASFA database in the Underlying Data section. Table 1 in our manuscript also provides a statistical description of the data used. Therefore, the discussion will focus more on the model variables rather than on the dairy system characteristics.
4. **Water Consumption and Greenhouse Gases Calculation:** We will include a detailed description of how water consumption and greenhouse gas emissions were calculated.
5. **Grazing Management and Other Characteristics:** Although detailed information on grazing management, forage resources, and breeds is available in the aforementioned repositories, we will ensure that the discussion incorporates relevant points to explain the results in the inefficiency model more effectively.

Thank you once again for your constructive suggestions. We will incorporate these revisions to enhance the clarity and support of our article.

 Best regards,

 Carlos

# Reviewer report: Reviewer response for version 2

**DOI:** 10.5256/f1000research.148751.r239237

**Published:** 2024-02-19

**Authors:** Jakub Staniszewski

**Affiliations:** 1 Uniwersytet Ekonomiczny w Poznianiu, Poznań, Poland

Dear Authors. General idea for the paper seems interesting, but your application requires some serious improvements.

1. Regarding the methodological part of the paper, authors refer to the well known SFA model Battese & Coelli 1995, which is intended for panel data. Also all the equations include it subscripts which would suggest a panel setting of the data. However, authors apply the model to cross-sectional data. For this type of data check sfaR package in R [1]
2. Authors introduce number of dependents and age of the producer as a production factors which has no theoretical justification. In the most of the application set of production factors is limited to labour, capital and land. Variables ND and EP are more suitable as explanatory variables in inefficiency component. Authors introduce also I and j subscripts for some variables but don't explain it.  
3. The way how environmental component was introduced into the model is completely incorrect. In this setting environmental pressure generated by the farms is not a part of production process which is an obvious error. For the ways of introducing environmental component into efficiency analysis see this review [2].
4. From the practical point of view 102 observations is quite little for SFA model. I would rather implement DEA approach. 
5. Regarding the presentation of the results. Efficiency scores are usually presented on density plot not linear trend. Also a full table with production function model parameters and their significance is missing. 
6. I don't understand why authors used geometric mean either. 
7. Regarding conclusions, they cannot be right as they are based on flawed model.
8. To sum up, authors collected interesting data, but their empirical application has some serious drawbacks 

Is the work clearly and accurately presented and does it cite the current literature?

No

If applicable, is the statistical analysis and its interpretation appropriate?

No

Are all the source data underlying the results available to ensure full reproducibility?

Yes

Is the study design appropriate and is the work technically sound?

No

Are the conclusions drawn adequately supported by the results?

No

Are sufficient details of methods and analysis provided to allow replication by others?

Yes

Reviewer Expertise:

environmentally adjusted efficiency analysis

I confirm that I have read this submission and believe that I have an appropriate level of expertise to state that I do not consider it to be of an acceptable scientific standard, for reasons outlined above.

## References

[1] : ENVIRONMENTALLY ADJUSTED ANALYSIS OF AGRICULTURAL EFFICIENCY: A SYSTEMATIC LITERATURE REVIEW OF FRONTIER APPROACHES. *Zagadnienia Ekonomiki Rolnej / Problems of Agricultural Economics* .2023;374(1) : 10.30858/zer/162644 20-41 10.30858/zer/162644

[2] : Stochastic Frontier Analysis Routines.2023;

# Response

**Published:** 2024-06-08

**Authors:** C. A. Zuniga-Gonzalez

**Affiliations:** Agroecology. ECAV. CIByCC., National Autonomous University of Nicaragua, Leon. Bioeconomy and Climate Change Research Center., Leon, Leon, Nicaragua

Response to the reviewer # 1 Jakub Staniszewski, Uniwersytet Ekonomiczny w Poznianiu, Poznań, Poland

 Dear Authors. General idea for the paper seems interesting, but your application requires some serious improvements.

 Reviewer # 1

 [1] Regarding the methodological part of the paper, authors refer to the well known SFA model Battese & Coelli 1995, which is intended for panel data. Also all the equations include it subscripts which would suggest a panel setting of the data. However, authors apply the model to cross-sectional data. For this type of data check sfaR package in R [1].

 Response to Reviewer # 1

 Dear Reviewer, Thank you very much for your valuable feedback on our manuscript. We appreciate your careful review and constructive comments. We have revised the methodological section of the paper to align with your suggestions. Specifically, we have corrected the equations and the text to accurately reflect that our study is based on cross-sectional data rather than panel data. The subscripts indicating time have been removed, and the description of the variables and the model have been adjusted accordingly. Thank you once again for your insightful comments, which have helped improve the clarity and accuracy of our manuscript.

 Reviewer # 1

 [2] Authors introduce number of dependents and age of the producer as a production factors which has no theoretical justification. In the most of the application set of production factors is limited to labour, capital and land. Variables ND and EP are more suitable as explanatory variables in inefficiency component. Authors introduce also I and j subscripts for some variables but don't explain it. 

 Response to Reviewer # 1

 Dear Reviewer, Thank you very much for your valuable feedback on our manuscript. We appreciate your careful review and constructive comments.

 Regarding your concern about the inclusion of the number of dependents (ND) and the age of the producer (EP) as production factors, we would like to provide a theoretical justification for their inclusion in the production function (equation 4).

 Age of the Producer (EP): The age of the producer is considered a proxy for the experience and accumulated knowledge that can enhance management practices and decision-making in dairy farming. Experienced producers are likely to be more efficient in utilizing resources and implementing effective production techniques, which can directly impact productivity.

 Number of Dependents (ND): In the context of family-run dairy farms, the number of dependents often correlates with the availability of additional family labor. This labor can be crucial in small-scale operations where family members contribute significantly to farm activities, thereby influencing overall productivity.

 We have clarified this theoretical justification in the revised manuscript to reflect the rationale behind including these variables as part of the production function.

 Additionally, we have corrected the subscripts and ensured that the methodological section accurately reflects the cross-sectional nature of our data.

 Thank you once again for your insightful comments, which have significantly contributed to improving the quality of our manuscript.

 Here is the theoretical justification:

 Reviewer # 1

 [3]The way how environmental component was introduced into the model is completely incorrect. In this setting environmental pressure generated by the farms is not a part of production process which is an obvious error. For the ways of introducing environmental component into efficiency analysis see this review.

 Response to Reviewer # 1

 Thank you for your thorough review and valuable feedback on our manuscript. We appreciate your insights and the opportunity to clarify our methodology.

 Response to the Comment on Environmental Component Integration

 Regarding your concern about the integration of the environmental component into our model, we respectfully disagree with your assertion that the environmental pressure generated by the farms is not a part of the production process.

 In our study, we aimed to capture the comprehensive impact of dairy farming, which includes both economic production and environmental consequences. The variables related to environmental pressure, such as greenhouse gas emissions (CH4) and water use per animal unit (CAE), are critical to understanding the overall efficiency and sustainability of the dairy production process.

 Theoretical Justification for Environmental Variables

 Greenhouse Gas Emissions (CH4): Methane emissions from enteric fermentation represent a significant environmental impact of dairy farming. Including CH4 in the inefficiency model allows us to account for the environmental pressures and their influence on production efficiency. By doing so, we acknowledge that environmental factors are intertwined with the production process and can affect the overall performance of the farm.

 Amount of Water Used per Animal Unit (CAE): Efficient water use is vital in dairy farming, particularly in areas where water scarcity can limit production. The amount of water used per animal unit reflects the farm's resource efficiency and can significantly impact productivity and environmental sustainability. Incorporating CAE in the inefficiency model aligns with our objective to evaluate how environmental resource management affects production efficiency.

 Our approach is supported by the literature, which emphasizes the importance of considering environmental factors in efficiency analysis. For instance, several studies have integrated environmental variables into stochastic frontier analysis to assess their impact on technical efficiency.

 Clarification of the Model

 To further clarify, our model does not treat environmental pressures as direct inputs in the production function but rather as factors influencing the inefficiency component. This distinction is crucial, as it allows us to evaluate how well farms manage both their production and their environmental impacts.

 Reviewer # 1

 [4] From the practical point of view 102 observations is quite little for SFA model. I would rather implement DEA approach.

 Response to Reviewer # 1

 Dear Reviewer, Thank you for your thorough review and valuable feedback on our manuscript. We appreciate your insights and the opportunity to address your concerns.

 Response to the Comment on the Sample Size and Methodology

 Regarding your comment on the sample size and the suggestion to use the DEA approach instead of the SFA model, we would like to provide the following clarifications and justifications:

 Sample Size Consideration

 While it is true that a larger sample size is generally preferred for robust statistical analysis, the application of the Stochastic Frontier Analysis (SFA) with 102 observations is still valid and has been documented in the literature. Several studies have successfully applied SFA with relatively small sample sizes, demonstrating its flexibility and applicability in various contexts.

 Justification for Using SFA Over DEA

 Statistical Noise Handling: One of the primary advantages of SFA over DEA is its ability to separate inefficiency from statistical noise. SFA incorporates a stochastic error term, which allows it to account for random shocks and measurement errors that may affect the production process. This is particularly important in our study, where environmental factors such as weather conditions can introduce variability that is beyond the control of the farmers.

 Parametric Nature: SFA is a parametric approach, which means it specifies a functional form for the production frontier. This allows us to make more precise inferences about the production technology and the relationship between inputs and outputs. In contrast, DEA is a non-parametric method and does not impose a specific functional form, which can lead to less precise efficiency estimates in the presence of noise.

 Integration of Environmental Variables: Our study aims to incorporate environmental variables such as greenhouse gas emissions (CH4) and water use per animal unit (CAE) into the efficiency analysis. SFA is well-suited for this purpose because it allows for the inclusion of these variables in the inefficiency model, providing insights into how environmental pressures impact production efficiency. DEA, while useful for certain applications, does not easily accommodate the integration of such explanatory variables in the same comprehensive manner.

 Literature Support: The use of SFA with smaller sample sizes is supported by several studies in agricultural economics and related fields. These studies demonstrate that SFA can yield meaningful and reliable results, even with a limited number of observations, provided the model is appropriately specified and the assumptions are met.

 Conclusion

 In conclusion, while we acknowledge the importance of sample size in statistical analysis, we believe that the application of the SFA model in our study is justified and appropriate given the context and objectives of our research. The SFA model's ability to handle statistical noise and incorporate environmental variables makes it a suitable choice for analyzing the efficiency of dairy farms in our dataset.

 We appreciate your suggestion to consider DEA, but we believe that SFA offers a more robust and insightful framework for our specific research goals.

 Reviewer # 1

 [5]Regarding the presentation of the results. Efficiency scores are usually presented on density plot not linear trend. Also a full table with production function model parameters and their significance is missing.

 Response to Reviewer # 1

 Dear Reviewer, Thank you for your insightful comments and suggestions regarding the presentation of our results. We appreciate your feedback and have taken steps to address the points you raised.

 Presentation of Efficiency Scores

 We agree that a density plot is more appropriate for presenting efficiency scores. We have replaced the linear trend plot with a density plot to better illustrate the distribution of the efficiency scores. This change provides a clearer visualization of the data.

 Inclusion of Model Parameters Table

 We acknowledge the importance of providing a full table with the production function model parameters and their significance. We have now included a comprehensive table in the manuscript, detailing the estimated coefficients, standard errors, t-values, and p-values for the model parameters. This addition enhances the transparency and interpretability of our results.

 The revised manuscript with these updates is attached for your review. We believe these changes address your concerns and improve the overall presentation of our findings.

 Reviewer # 1

  [6] I don't understand why authors used geometric mean either.

 Response to Reviewer # 1

 Dear Reviewer, Thank you for your continued feedback and for giving us the opportunity to clarify our methodology.

 Regarding your concern about the use of the geometric mean, we would like to explain the rationale behind this choice. In efficiency analysis, it is standard practice to use the geometric mean rather than the arithmetic mean to calculate average efficiency scores. This is because efficiency scores are typically multiplicative rather than additive, and the geometric mean provides a more accurate measure of central tendency for these types of data.

 Geometric Mean Justification:

 Nature of Efficiency Scores: Efficiency scores, by their nature, are ratios and often exhibit a skewed distribution. The geometric mean is more appropriate for such data because it minimizes the impact of extreme values, providing a more robust measure of central tendency.

 Multiplicative Data: In contexts where data are multiplicative, as is the case with efficiency scores, the geometric mean is more meaningful. It accurately reflects the central tendency of ratios and percentages, which are inherently multiplicative.

 Precedent in Literature: The use of geometric mean in efficiency analysis is well-documented and widely accepted in the literature. It ensures that the aggregated efficiency measure remains within the feasible range (i.e., between 0 and 1 for efficiency scores).

 By using the geometric mean, we aim to present a more reliable and interpretable measure of average efficiency, which aligns with best practices in the field of efficiency and productivity analysis.

 We hope this clarifies our approach. We appreciate your insights and are open to further discussion to improve our work.

 Reviewer # 1

  [7] Regarding conclusions, they cannot be right as they are based on flawed model.

 Response to Reviewer # 1

 Regarding the concern that our conclusions might be flawed due to an allegedly defective model, we respectfully disagree. We have thoroughly justified our model choice and the inclusion of environmental variables, as explained in our previous responses. The use of the geometric mean for efficiency scores and the integration of environmental factors into the inefficiency model are well-supported by existing literature and best practices in efficiency analysis.

 Our conclusions are based on robust statistical methods and significant results, demonstrating the reliability and relevance of our findings. We are confident that our model accurately captures the complexities of dairy farm efficiency and the impact of environmental factors.

 We appreciate your insights and hope that our explanations clarify the robustness of our model and the validity of our conclusions.

 Reviewer # 1

 [8] To sum up, authors collected interesting data, but their empirical application has some serious drawbacks

 Response to Reviewer # 1

 Dear Reviewer, Thank you for your thorough review and for recognizing the value of the data we collected. We appreciate your feedback and the opportunity to address your concerns.

 Response to the Comment on Empirical Application

 We acknowledge that the empirical application of our study has some limitations, as you have pointed out. However, we believe that the methodology we employed—Stochastic Frontier Analysis (SFA)—is appropriate for the objectives of our research. We have carefully considered the integration of environmental factors into the inefficiency model and provided a detailed justification for our approach.

 Addressing Specific Drawbacks

 Model Selection: We chose SFA over DEA because SFA allows us to incorporate statistical noise and provides a way to separate inefficiency from random error. This is particularly important in agricultural studies where external factors such as weather conditions can significantly affect production.

 Geometric Mean: The use of the geometric mean to report efficiency scores is a standard practice in efficiency analysis. It provides a more accurate representation of central tendency when dealing with ratio data, such as efficiency scores, which are bound between 0 and 1.

 Environmental Variables: Incorporating environmental variables like methane emissions (CH4) and water usage (CAE) into the inefficiency model is critical for understanding the broader impacts of dairy farming. These variables are not just peripheral but integral to assessing the sustainability and overall efficiency of agricultural practices.

 Moving Forward

 We are committed to further refining our empirical approach in future research. Your insights have been invaluable in highlighting areas for potential improvement. Specifically, we will consider exploring additional methodologies and robustness checks to enhance the reliability of our findings.

 Conclusion

 In conclusion, while there are always areas for improvement, we believe that our study provides significant insights into the efficiency and sustainability of dairy farming in Tlaxcala. We appreciate your constructive feedback, which will help us to strengthen our research and its contributions to the field.

 Finally, Dear Reviewer, Thank you for your valuable feedback and for providing the references to enhance our manuscript. We appreciate your insights and the opportunity to improve our work.

 Acknowledgment of References

 We have carefully reviewed the references you suggested:

 [1] Staniszewski J, Matuszczak A: "Environmentally Adjusted Analysis of Agricultural Efficiency: A Systematic Literature Review of Frontier Approaches." Zagadnienia Ekonomiki Rolnej / Problems of Agricultural Economics. 2023; 374 (1): 20-41.

 [2] Dakpo, H., Desjeux, Y., Henningsen, A., Latruffe, L. : "Stochastic Frontier Analysis Routines." 2023. https://cran.r-project.org/web/packages/sfaR/sfaR.pdf

 These references have been incorporated and cited in our revised manuscript. Their insights have helped us to better frame our methodology and contextualize our findings within the broader literature on agricultural efficiency and environmental impacts.

 Confirmation of Inclusion

 The references have been added to both the introduction and the methodology sections of our paper. They provide a robust foundation for our approach and support the integration of environmental factors into our efficiency analysis.

 Thank you once again for your constructive suggestions and for pointing us toward these valuable resources. We believe that their inclusion significantly strengthens our study.
